# Favorable outcomes in locally advanced and node positive prostate cancer patients treated with combined pelvic IMRT and androgen deprivation therapy

**DOI:** 10.1186/s13014-015-0540-3

**Published:** 2015-11-17

**Authors:** Wolfgang Lilleby, Amol Narrang, Gunnar Tafjord, Ljiljana Vlatkovic, Kjell Magne Russnes, Andreas Stensvold, Knut Håkon Hole, Phuoc Tran, Karsten Eilertsen

**Affiliations:** Department of Oncology, Oslo University Hospital, The Norwegian Radium Hospital, 0424 Oslo, Norway; Departments of Radiation Oncology and Molecular Radiation Sciences, Oncology and Urology, Johns Hopkins Hospital, Baltimore, MD USA; Department of Pathology, Oslo University Hospital, The Norwegian Radium Hospital, 0424 Oslo, Norway; Department of Oncology, Østfold Hospital Trust, 1603 Fredrikstad, Norway; Department of Radiology, Oslo University Hospital, The Norwegian Radium Hospital, 0424 Oslo, Norway; Department of Medical Physics, Oslo University Hospital, The Norwegian Radium Hospital, 0424 Oslo, Norway

**Keywords:** Intensity modulated radiotherapy, Prostate cancer, Pelvic lymph node

## Abstract

**Background:**

The most appropriate treatment for men with prostate cancer and positive pelvic nodes, N+, is an area of active controversy. We report our 5-years outcomes in men with locally advanced prostate cancer (T1-T4N0-N1M0) treated with definitive radiotherapy encompassing the prostate and pelvic lymph nodes (intensity modulated radiotherapy, IMRT) and long-term androgen deprivation therapy (ADT).

**Material and methods:**

Of the 138 consecutive eligible men all living patients have been followed up to almost 5 years. Survival endpoints for 5-year biochemical failure-free survival (BFFS), relapse-free survival (RFS), prostate cancer-specific survival (PCSS), and overall survival (OS) were assessed by Kaplan-Meier analysis. Univariate and multivariate Cox regression proportional hazards models were constructed for all survival endpoints. The RTOG morbidity grading system for physician rated toxicity was applied.

**Results:**

Patients with locally advanced T3-T4 tumors (35 %) and N1 (51 %) have favorable outcome when long-term ADT is combined with definitive radiotherapy encompassing pelvic lymph nodes. The 5-year BFFS, RFS, PCSS and OS were 71.4, 76.2, 94.5 and 89.0 %, respectively. High Gleason sum (9–10) had a strong independent prognostic impact on BFFS, RFS and OS (*p* = 0.001, <0.001, and 0.005 respectively). The duration of ADT (= > 28 months) showed a significant independent association with improved PCSS (*p* = 0.02) and OS (*p* = 0.001). Lymph node involvement was not associated with survival endpoints in the multivariate analysis. The radiotherapy induced toxicity seen in our study population was moderate with rare Grade 3 GI side effects and up to 11 % for Grade 3 GU consisting mainly of urgency and frequency.

**Conclusion:**

Pelvic IMRT in combination with long-term ADT can achieve long-lasting disease control in men with N+ disease and unfavorable prognostic factors.

**Electronic supplementary material:**

The online version of this article (doi:10.1186/s13014-015-0540-3) contains supplementary material, which is available to authorized users.

## Introduction

Optimal treatment of locally advanced and lymph node-positive (cN1 or pN1) adenocarcinoma of the prostate has not yet been determined. There is abundant evidence gained from phase III studies that a substantial number of patients with locally advanced prostate cancer (PCa) derive a survival benefit from the combination of radiation and hormonal therapy [1–4]. Randomized trials of elective nodal irradiation of the pelvic lymph nodes in N0 men have not been shown to confer a survival benefit in similar patients [5, 6]. Therefore, the most appropriate treatment for men with clinically positive pelvic nodes, cN1 (or cN+), is an area of active controversy. The experience from extended lymphadenectomy suggests that for a subgroup of patients with limited positive pelvic lymph nodes, pN1, irradiation of the pelvic lymphatic structures could translate into long-lasting disease control [7, 8]. Similarly, there is emerging retrospective and prospective data that definitive radiation therapy and androgen deprivation therapy (ADT) in cN+ may be more beneficial than ADT alone [9–14].

In the present study we investigated the 5-year outcomes of patients with locally advanced and/or with N+ prostate cancer undergoing intensity modulated radiotherapy (IMRT) combined with long-term androgen deprivation therapy (ADT).

## Patients and methods

### Cohort characteristics

This study concerns consecutively treated patients treated with IMRT (*n* = 138) to the prostate, seminal vesicles and pelvic nodal basins and/or clinical positive nodal disease who had at least a 5-years of follow-up. Two patients were excluded from the survival analyses due to missing lymph node status. Only patients with less than 3 positive lymph nodes were eligible. The protocol was approved by the Ethics Committee of the Health Region South/East of Norway. All patients gave written consent.

### Nodal sampling and N+ criteria

In addition to the anatomical grading using the TNM/UICC stage classification [15], the inclusion criteria were: age < 75 years, no previous invasive cancer, initial PCa diagnosis made during the last 6 months, pN + M0 or a calculated N+ risk = > 15 % using the Memorial Sloan-Kettering Cancer Center nomogram [16] and prognostic high-risk disease defined by D’Amico’s classification [17]. During the recruitment period from 2004 to 2010 a trend towards radiographic N-assessment by magnetic resonance imaging (MRI) was seen due to low yield in the dissected lymph nodes obtained mainly from the obturator region when applying standard lymphadenectomy [18].

### IMRT planning and delivery

The delineation of the clinical target volume (CTV) and organs at risk has been described in detail previously [19]. Briefly, the CTV for the pelvic nodes was delineated by contouring a 0.7 cm radial area around the pelvic iliac vessels and adding a 2 mm margin to obtain a planning target volume (PTV). The medial portion of the presacral nodal area was left out in the delineation of lymph nodes, aiming to spare the recto-sigmoid, otherwise the contouring was per the recommendations attainable by the Radiation Therapy Oncology Group Web site (www.rtog.org).

The rectum was contoured from the anus to the rectosigmoid flexure. In the approved IMRT protocol predefined protocol-stated dose constraints to the OARs were mandatory. The use of 3D-conformal radiation therapy (CRT) and IMRT in prostate cancer permits dose escalation strategies with improved sparing of normal tissue.

The inverse planning software in Oncentra Masterplan (Nucletron, Veenendal, The Netherlands) was applied during the pilot phase of the study, and after 2006 the inverse planning software Konrad obtained from MRC Systems (MRC Systems GmbH, Heidelberg, Germany) was applied. Treatment plans were generated by seven coplanar fields to the delineated pelvic structures up to a total dose of 50 Gy encompassing the prostate, seminal vesicles and nodal basins (target volumes; PTV) by use of 15-MV photon beams. Radiation to the boost volume (24 Gy to the seminal vesicles and the prostate for T3b; 24 Gy to the prostate alone for ≤ T3a) was done by a four-field box technique. No attempt for dose escalation to N+ patients was included in this protocol. Patients were instructed to empty the rectum and keep the bladder filled during the course of radiotherapy. In the optimized plan the prescribed radiation dose was set equal to the mean dose of the ITV according to the International Commision on Radiation Units and Measurements Reports 62 (ICRU Report 62, www.icru.org). Image guided patient set-up and irradiation was performed by daily field matching on bony landmarks for the majority of patients and on three implanted fiducial markers (Goldlock III, BeamPoint AB, Stockholm, Sweden) in 30 patients.

### Androgen deprivation therapy (ADT)

All patients started neo-adjuvant ADT 6 months prior to IMRT, and this treatment was continued to a maximum of 2.5 years in some patients with pN+ and a very high-risk profile (Table 1). We applied a 3-months depot injection with gosereline (Zoladex® 10.8 mg sc). As prophylaxis against flare-ups, bicalutamide 50 mg × 1 orally was given for 30 days, beginning 1 week prior to first injection of gosereline.

**Table 1:** Demographic, disease, and treatment characteristics

Characteristic	Lymph node positive (*n* = 58)	Lymph node negative (*n* = 78)	*P*-value
Median age at diagnosis (range), yrs	66.7 (50.9 – 76.8)	67.4 (48.3 – 79.1)	0.03
Mode of detection, *n* (%)			0.17
Symptomatic	25 (43)	25 (32)	
Screening	33 (57)	53 (68)	
Median PSA (range), ng/mL	24.4 (1.8 – 109.0)	26.0 (2.9 – 109.0)	0.26
Gleason sum, *n* (%)			0.21
3 + 3	3 (5)	1 (1)	
3 + 4	10 (17)	8 (10)	
4 + 3	13 (22)	22 (28)	
4 + 4	23 (40)	29 (37)	
4 + 5	5 (9)	16 (21)	
5 + 4	3 (5)	2 (3)	
5 + 5	1 (2)	0 (0)	
Clinical stage, *n* (%)			0.58
T1b	1 (2)	0 (0)	
T1c	1 (2)	6 (8)	
T2a	3 (3)	1 (1)	
T2b	3 (5)	4 (5)	
T2c	3 (5)	3 (4)	
T3a	23 (40)	32 (41)	
T3b	23 (40)	30 (38)	
T4	1 (2)	2 (3)	
Median duration of ADT (range), months	40.3 (11.9 – 54.4)	28.7 (9.0 – 60.2)	<0.001

Abbreviations: prostate-specific antigen (*PSA*)

### Survivorship procedure

Patients were regularly seen in the outpatient clinic at 3–4 month intervals the first 2 years, and then every 6 months for the next 3 years. Routine history and physical exam was performed including blood samples with PSA and testosterone.

### Physician-rated toxicity at 36 months

Consecutively reported side effects or dysfunction on erectile potency, urinary, and bowel function were rated by one physician (WL) at regular intervals during follow-up. Grading of gastrointestinal and kidney-bladder side effects was done by the International Toxicity/RTOG morbidity grading scale [20] Patients’ self-rating side effects have been published previously [21], but here we only report the prevalence of Grade 2 or more toxicity observed during the study period.

### Statistical analysis

Primary endpoints of our study included biochemical failure-free survival (BFFS), relapse-free survival (RFS), prostate cancer-specific survival (PCSS), and overall survival (OS). Biochemical failure was defined in accordance with the Radiation Therapy Oncology Group – Association of Therapeutic Radiation Oncology (RTOG-ASTRO) Phoenix Consensus Conference definition. Relapse-free survival included local-regional or distant recurrence, as diagnosed by clinical exam, imaging, and/or biopsy. Death due to prostate cancer was defined as death in a patient with a documented history of hormone-refractory metastatic prostate cancer, evidence of a rising PSA at last follow-up visit, and no other obvious cause of death. Additionally, death certificates were cross-referenced to confirm cause of death. Patients who were alive were censored at last follow-up. For the purpose of calculating BFFS, patients without biochemical failure were censored at time of last PSA measurement. Survival endpoints were measured from the first day of initiation of ADT.

Differences in patient and treatment characteristics were compared between patients with and without radiologic and/or pathologic lymph node involvement using the *χ*^2^ test. Thresholds for categorical variables were defined in accordance with the literature. Univariate and multivariable Cox proportional hazards models were constructed for all survival endpoints. Hazard ratios for the associations between potential prognostic factors and survival endpoints were analyzed. Multivariable models adjusted for age at diagnosis, pre-treatment PSA, biopsy Gleason score, clinical stage, lymph node involvement, and duration of administration of ADT. Duration of ADT was calculated as the difference between the start and end dates of ADT administration, except when patients experienced biochemical failure, clinical recurrence, or death prior to completion of the originally prescribed course of ADT. In these cases, duration of ADT was calculated as the difference between the event date and start date of ADT. For each survival endpoint, backwards elimination was used to identify which covariates to include in the final multivariable model, using a threshold of *P*-value of 0.25 to make this determination. Additionally, Kaplan-Meier survival curves were constructed for all survival endpoints, with stratification by lymph node status and comparison of patient subsets using the log-rank test. Survival estimates at specific time points were derived from life tables.

Throughout the analysis, two-sided significance testing was used, and a *P*-value of 0.05 was considered statistically significant. All statistical analyses were performed with Stata software (Stata/IC10.0).

## Results

Demographic, tumor, and treatment characteristics are detailed in Table 1. The proportion of men with at least one high-risk factor (T3a or PSA >20 ng/mL or Gleason 8–10) or T3b-T4, Gleason grade 5 or two or more risk factors (very high-risk) were 25 and 75 %, respectively. Radiologic and/or pathologic lymph node evaluation was positive for 71 patients (51 %). Men with lymph node involvement were younger and more likely to receive a duration of ADT that was greater than 28 months, as compared to men without lymph node involvement (both *p* < 0.05). Mode of detection, initial PSA level, Gleason score, and clinical stage did not differ based on lymph node status.

Median follow-up of this cohort was 4.9 years (range: 0.9–11.1 years). Of the 136 men in the cohort who underwent combination treatment, 40 patients (29 %) experienced biochemical failure during the study period, and 34 patients (25 %) developed clinical relapse, including 15 patients (11 %) with distant metastases, 15 patients (11 %) with local-regional disease, and 4 patients (3 %) with concurrent distant and local-regional disease at time of relapse. In total, 18 patients (15 %) died during the follow-up period, including 8 patients (6 %) who died due to prostate cancer and 10 patients (7 %) who died from other causes.

**Fig. 1:**
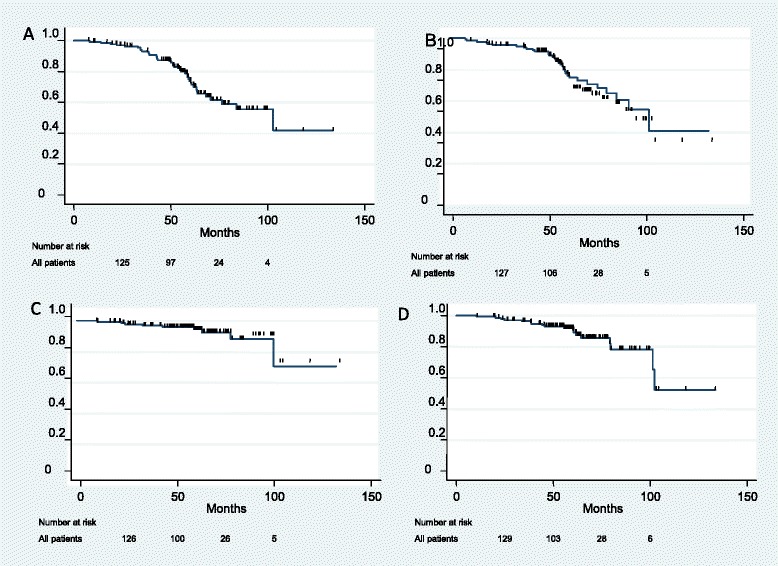
Kaplan-Meier survival estimates for **a** biochemical failure-free survival, **b** relapse-free survival, **c** prostate cancer-specific survival, and **d** overall survival for overall cohort

**Fig. 2:**
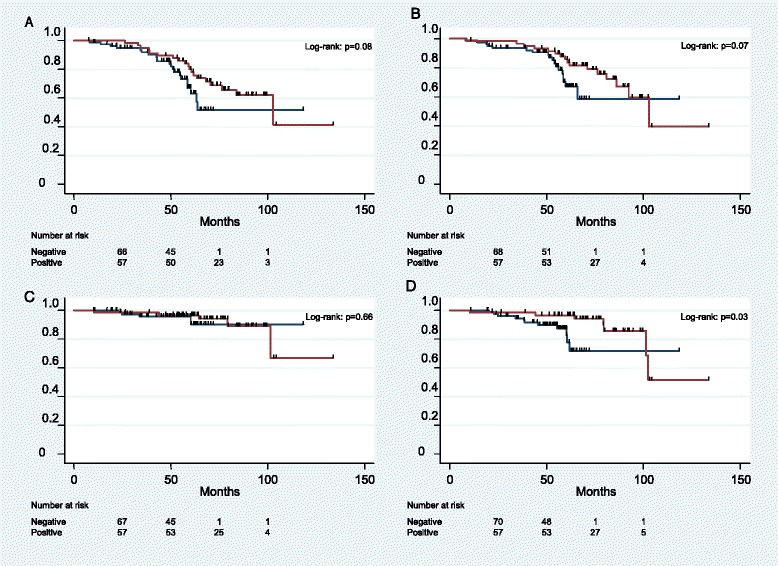
Kaplan-Meier survival estimates for **a** biochemical failure-free survival, **b** relapse-free survival, **c** prostate cancer-specific survival, and **d** overall survival, stratified by lymph node status. Red curves represent men with radiologic and/or pathologic lymph node involvement. Blue curves represent men without lymph node involvement

For the entire cohort, the 5-year BFFS and 5-year RFS were 71.4 and 76.2 % respectively. The 5-year PCSS and 5-year OS were 94.5 and 89.0 % respectively (Fig. 1). Figure 2 displays Kaplan-Meier estimates for survival endpoints, stratified by the presence of lymph node involvement. Men with lymph node positive disease experienced similar 5-year BFFS (*p* = 0.08), 5-year RFS (*p* = 0.07), and 5-year PCSS (*p* = 0.66), to those without pelvic nodal involvement. Interestingly, men with lymph node involvement experienced improved 5-year OS, as compared to men without lymph node involvement (96.5 % vs. 78.3 %, *p* = 0.03).

On univariate analysis, a higher Gleason score was associated with inferior BFFS (*p* = 0.001) and inferior RFS (*p* = 0.001), but was not associated with either PCSS or OS (Additional file 1: Table S1). Additionally, a higher clinical stage was associated with inferior BFFS (*p* = 0.03) and inferior RFS (*p* = 0.05), but not with PCSS or OS. Interestingly, while lymph node involvement was not associated with BFFS, RFS or PCSS, it was associated with improved OS (*p* = 0.03). A duration of ADT greater than 28 months was also associated with improved PCSS (*p* = 0.03) and improved OS (*p* < 0.001) on univariate analysis.

Table 2 details adjusted hazard ratios for the covariates included in the multivariable model, displaying results for covariates with a *p*-value of ≤0.10. A Gleason sum of 9 or 10 was associated with inferior BFFS (*p* = 0.001), RFS (*p* < 0.001), and OS (*p* = 0.005), and had a borderline statistically significant association with PCSS (*p* = 0.07). Additionally, a duration of ADT greater than 28 months had a statistically significant association with improved PCSS (*p* = 0.02) and improved OS (*p* = 0.001). Lymph node involvement was not associated with any of the survival endpoints on multivariate analysis.

**Table 2:** Multivariate Cox proportional hazards analysis for associations between covariates and survival endpoints

Covariate	Hazard ratio (95 % confidence interval)	*P*-value
*Biochemical failure-free survival*		
Gleason sum 9 or 10	3.33 (1.62 – 6.85)	0.001
Clinical stage T3b/T4	2.17 (1.11 – 4.22)	0.02
*Relapse-free survival*		
Gleason sum 9 or 10	4.43 (2.14 – 9.14)	<0.001
*Prostate cancer-specific survival*		
Gleason sum 9 or 10	4.13 (0.87 – 19.58)	0.07
Duration of ADT ≥28 months	0.20 (0.05 – 0.81)	0.02
*Overall survival*		
Age ≥65 years	3.57 (1.00 – 12.82)	0.05
Gleason 4 + 3	17.26 (1.11 – 268.69)	0.04
Gleason 4 + 4	14.67 (1.07 – 200.81)	0.04
Gleason sum 9 or 10	70.11 (3.49 – 1407.99)	0.005
Duration of ADT ≥28 months	0.15 (0.05 – 0.44)	0.001

Multivariable models adjusted for age at diagnosis, pre-treatment PSA, biopsy Gleason score, clinical stage, lymph node involvement, and duration of administration of ADT

Abbreviations: androgen deprivation therapy (*ADT*)

Threshold of *p* < 0.25 was used to exclude covariates from the final multivariable model, using backwards elimination

Maximum genitourinary and gastrointestinal toxicities are shown in Table 3. During the follow-up period, urinary frequency and urinary urgency were the most common grade 2 or higher genitourinary toxicities experienced by patients, with 10 % of patients experiencing grade 3 frequency and 11 % of patients experiencing grade 3 urgency. The most common grade 2 gastrointestinal toxicities were fecal urgency and blood in the stool, but grade 3 gastrointestinal toxicities were rare overall.

**Table 3:** Prevalence of RTOG grade 2 or higher genitourinary or gastrointestinal toxicity

	RTOG Grade 2 toxicity	RTOG Grade 3 toxicity
GU-tract, *N* (%)		
Hematuria	2 (1)	1 (<1)
Urinary retention	6 (4)	2 (1)
Urinary frequency	49 (36)	14 (10)
Dysuria	5 (4)	2 (1)
Urinary urgency	63 (46)	15 (11)
Urinary leakage	5 (4)	1 (<1)
GI-tract, *N* (%)		
Fecal urgency	37 (27)	2 (1)
Blood in stool	17 (13)	0 (0)
Loose stools	10 (7)	1 (<1)
Fecal incontinence	10 (7)	1 (<1)

Abbreviations: Genitourinary (*GU*), Gastrointestinal (*GI*)

## Discussion

In our study of locally advanced and N+ men with prostate cancer, we found a low risk for prostate-specific mortality (<6 %) during a median of 4.9-years of follow-up, but moderate biochemical and/or clinical relapse with combined treatment of pelvic IMRT and ADT for the entire cohort. This agrees with the efficacy of combination treatment as shown in pivotal phase III trials for locally advanced PCa [1–4].

Our results in a cohort where 51 % of men were N+ are very favorable at 5 years when compared to the aforementioned phase III trials for locally advanced, but predominantly N0 PCa, On multivariable analysis, we did not find nodal involvement to be prognostic. Furthermore, in our cohort we found an interesting observation whereby men with N+ had an improved overall survival compared to patients without lymph node involvement. In the latter, duration of ADT was an independent predictor for survival. We did identify Gleason sum of 9–10 as a universally independent poor prognostic factor for all our clinical endpoints. However, long-term duration of ADT, greater than 28 months, was found to be an independent favorable prognostic factor for PSCC and OS. This last finding is in agreement with level 1 evidence from several randomized trials [1, 3, 4, 22] that long-term/life-long ADT should be applied in men with locally advanced PCa who are treated with radiotherapy.

Until recently, N+ has been synonymous with M1 disease, with most men being treated routinely with indefinite ADT alone and no local therapy. However, patients with M1 disease have a deleterious 5 year relative survival of 35 % underpinned by the latest report of the Norwegian Cancer Registry (www.kreftregisteret.no). All of the prospective radiotherapy trials that recruited men with locally advanced PCa involved none or a minority of N+ patients (<5 %). Recently population based studies have suggested that the addition of local therapy in the N1 setting can have profound effects on survival [13, 14, 23]. Specifically, the addition of radiation to cN1 patients has suggested these patients may enjoy long-term disease control [24]. However, the optimal duration of ADT in men with N1 disease who receive combined radiotherapy and ADT is unknown. Longer-term ADT, but not indefinite ADT, was able to overcome the prognostic significance of N+ status in our cohort (Table 3). Our data suggests that indefinite ADT may not be needed for limited N1 patients when combined with radiotherapy. In addition, dose escalation exceeding 50 Gy to cN1 pelvic lymph nodes not approached in our protocol may translate in long-lasting disease control.

Our study population consists of patients with aggressive tumors. Micrometastatic disease at diagnosis not detected by today’s staging methods in these patients could have been associated with early relapse [25]. However, rare detection of disseminated tumor cells in the bone marrow (data not shown) and limiting eligibility to < 3 positive LNs supports our assumption that patients included in this study were not out of range for curatively intended treatment. Moreover, in these patients a major aim is to achieve local control by eradication of the primary tumor with combined treatment and thereby preventing re-seeding of tumor cells [26].

Involving additional pelvic structures into the prostate irradiation fields increases the possibility for toxicity. The prevalence of grade 2 to 3 GI and or GU toxicity was moderate and mainly related to urgency. The rare event of grade 3 rectal bleeding might be due to early referring of patients to laser coagulation or hyperbaric oxygenation as a coping strategy. Our recently published patient self-reported results showed only a moderate increase of GI/GU side-effects not affecting overall QoL [19, 21]. Many of the men on this trial where treated before strict measures for daily image guidance were institutionalized, such as the use of fiducial markers in combination with Cone-Beam-CT. Such a patient set-up verification procedure allows a reduction of the treatment margins related to organ motion and set-up uncertainties, and a reduction in toxicity can thereby be expected. In addition, strategies addressing systemic treatments with a lower toxicity profile such as therapeutic cancer vaccines or novel class of androgen receptor inhibitors substituting partly ADT should be investigated [27, 28].

### Strengths and limitations

Our study limitations include retrospective analyses and the lack of randomization. Still, the similar work-up regimen and homogeneous treatment applications in this cohort of men with locally advanced PCa and/or N1 disease generated some interesting findings which are clinically meaningful in the wake of future results from randomized trials in this risk group.

## Conclusion

Men with locally advanced and/or N1 prostate cancer have favorable outcomes following combined radiation and ADT and this can be achieved with low levels of toxicity when using IMRT to pelvic structures.

## Additional file

Additional file 1: Table S1. Univariate Cox proportional hazards analysis for associations between covariates and survival endpoints. (DOC 48 kb)
