# Supplementary material for: Favorable outcomes in locally advanced and node positive prostate cancer patients treated with combined pelvic IMRT and androgen deprivation therapy
Source: Radiat Oncol. 2015 Nov 17;10:232. doi: 10.1186/s13014-015-0540-3 (PMC4650510; doi:10.1186/s13014-015-0540-3)
Supplement: Additional file 1: Table S1. — Univariate Cox proportional hazards analysis for associations between covariates and survival endpoints. (DOC 48 kb) [file 13014_2015_540_MOESM1_ESM.doc]

Supplemental etable 1: Univariate Cox proportional hazards analysis for associations between covariates and survival endpoints

| Covariate | **Hazard ratio (95% confidence interval)** | **P-value** |
| --- | --- | --- |
| *Biochemical failure-free survival* | | |
| Age ≥65 years | 0.59 (0.32 – 1.11) | 0.10 |
| Initial PSA in ng/mL, n (%)  <10  10-20  >20 | ---  0.29 (0.29 – 2.99)  1.07 (0.37 – 3.09) | 0.91  0.91 |
| Gleason sum, n (%)  3+3/3+4  4+3  4+4  Gleason sum 9-10 | ---  2.31 (0.62 – 8.56)  2.62 (0.75 – 9.13)  9.09 (2.58 – 32.01) | 0.21  0.13  0.001 |
| Clinical stage  T1-T2  T3a  T3b/T4 | ---  1.58 (0.52 – 4.77)  3.23 (1.09 – 9.56) | 0.42  0.03 |
| Lymph node involvement | 0.56 (0.29 – 1.08) | 0.09 |
| Duration of ADT ≥28 months | 0.88 (0.43 – 1.80) | 0.72 |
| *Relapse-free survival* | | |
| Age ≥65 years | 0.66 (0.33 – 1.32) | 0.24 |
| Initial PSA in ng/mL, n (%)  <10  10-20  >20 | ---  0.66 (0.20 – 2.22)  0.81 (0.27 – 2.40) | 0.50  0.71 |
| Gleason sum, n (%)  3+3/3+4  4+3  4+4  Gleason sum 9-10 | ---  2.54 (0.51 – 12.74)  3.75 (0.81 – 17.31)  15.21 (3.34 – 69.26) | 0.26  0.09  0.001 |
| Clinical stage  T1-T2  T3a  T3b/T4 | ---  1.71 (0.49 – 6.01)  3.48 (1.01 – 12.00) | 0.40  0.05 |
| Lymph node involvement | 0.51 (0.25 – 1.07) | 0.08 |
| Duration of ADT ≥28 months | 0.67 (0.33 – 1.39) | 0.29 |
| *Prostate cancer-specific survival* | | |
| Age ≥65 years | 1.12 (0.28 – 4.44) | 0.87 |
| Initial PSA in ng/mL, n (%)  <10  10-20  >20 | ---  0.39 (0.05 – 2.80)  0.53 (0.10 – 2.89) | 0.35  0.47 |
| Gleason sum^a^, n (%)  4+3  4+4  Gleason sum 9-10 | ---  1.34 (0.24 – 7.43)  3.69 (0.59 – 22.99) | 0.74  0.16 |
| Clinical stage  T1-T2  T3a  T3b/T4 | ---  0.52 (0.07 – 3.73)  2.22 (0.37 – 13.47) | 0.52  0.39 |
| Lymph node involvement | 0.73 (0.17 – 3.08) | 0.66 |
| Duration of ADT ≥28 months | 0.23 (0.06 – 0.87) | 0.03 |
| *Overall survival* | | |
| Age ≥65 years | 1.29 (0.49 – 3.41) | 0.60 |
| Initial PSA in ng/mL, n (%)  <10  10-20  >20 | ---  0.47 (0.10 – 2.16)  0.68 (0.19 – 2.50) | 0.33  0.56 |
| Gleason sum^a^, n (%)  4+3  4+4  Gleason sum 9-10 | ---  0.84 (0.25 – 2.78)  2.85 (0.84 – 9.59) | 0.77  0.09 |
| Clinical stage  T1-T2  T3a  T3b/T4 | ---  0.46 (0.12 – 1.86)  1.59 (0.49 – 5.20) | 0.28  0.44 |
| Lymph node involvement | 0.31 (0.11 – 0.90) | 0.03 |
| Duration of ADT ≥28 months | 0.17 (0.06 – 0.45) | <0.001 |

Abbreviations: prostate-specific antigen (PSA); androgen deprivation therapy (ADT)

Multivariable models adjusted for age at diagnosis, pre-treatment PSA, biopsy Gleason score, clinical stage, lymph node involvement, and duration of administration of ADT

^a^Dummy variable representing Gleason 3+3 disease was excluded from analysis with PCSS and OS as endpoints, due to lack of events in these patients
